# Introduction

**DOI:** 10.1007/978-3-030-46636-7_1

**Published:** 2020-06-04

**Authors:** Terence McNamee, Monde Muyangwa

**Affiliations:** 1 Global Fellow, Africa Program, Woodrow Wilson International Center for Scholars, Washington, DC USA; 2 Director, Africa Program, Woodrow Wilson International Center for Scholars, Washington, DC USA; 3 Global Fellow, Africa Program, Woodrow Wilson International Center for Scholars, Washington, DC USA; 4 Director, Africa Program, Woodrow Wilson International Center for Scholars, Washington, DC USA

## Abstract

The introduction briefly summarizes the thematic chapters in the book (conflict prevention, mediation and management; post-conflict reconstruction, justice and DDR; the role of women, religion, humanitarianism, grassroots organizations and early warning systems; and regional and continental bodies) as well as the country/region case studies (the Democratic Republic of Congo, Rwanda, Sierra Leone, Sudan/South Sudan, Mozambique and the Sahel/Mali). The introduction also outlines the key conceptual and definitional challenges and explains what sets this volume apart from others in the ever-expanding literature on peacebuilding in Africa. Of several recurrent themes in the book that merit closer scrutiny, the introduction highlights: funding challenges; managing expectations; tensions between grassroots dynamics and peace-building at the elite level; varying effectiveness of regional economic communities and the African Union; and frequent lack of coordination between donors and partners on the ground.

The birth year of the Organization of African Unity (OAU), 1963, is often considered Africa’s year of independence. But political freedom did not mean freedom from the repression and violence which had characterized the colonial period. Wars and conflicts have scarred the continent since independence. After the fall of the Berlin Wall in 1989, they became more complex and widespread. And so, too, did the international efforts to restore and (re) build peace in Africa. Countries worst affected by violence and conflict included Sierra Leone, Liberia, Rwanda, Somalia, the Democratic Republic of the Congo, Sudan/South Sudan, Central African Republic, Mali, and Libya. In recent years, the quest for sustainable peace in Africa has taken on a new urgency, as instability and insecurity continue to negatively impact the lives of millions of Africans and hinder the continent’s economic growth and development. This book joins the quest for peace by examining 30 years of peacebuilding in Africa, highlighting key lessons learned and offering some recommendations for making peace stick.

In 2013, the Heads of State and Governments of the African Union (AU) signed the 50th Anniversary Solemn Declaration. To mark a half-century since the formation of the OAU, forerunner to the AU, leaders committed to work for peace and prosperity and end strife on the continent by 2020.

Our determination to achieve the goal of a conflict-free Africa, to make peace a reality for all our people, and to rid the continent of wars, civil conflicts, human rights violations, humanitarian disasters, and violent conflicts, and to prevent genocide, We pledge not to bequeath the burden of conflicts to the next generation of Africans, and undertake to end all wars in Africa by 2020.^1^

Presumably, none of the signatories genuinely believed that such an ambitious target could be achieved within seven years. In the period since the founding of the OAU, more than half of African states experienced some form of major conflict. Many of those states had reverted to war after periods of relative peace. The signatories knew that. Most were in their 60s or older in 2013; they had lived through some of Africa’s bleakest times. Today, the continent cannot be described as “conflict-free.”

At the same time, Africa has progressed further down the path of peace than is typically portrayed. State fragility remains an endemic problem across the continent, but incidents of mass violence are increasingly rare. This is in no small part due to African states shedding the tactics of their former colonial masters and taking ownership of the problems within their own borders. As one of our contributors observes, Africa has

…exhibited a notable readiness to assume the tasks of crisis management and engage in mutual cooperation between states to rebuild stability, through diplomacy, negotiation and the deployment of intervention forces and peacekeepers. The continent’s capacity for common action is one of its greatest strengths.^2^

To add weight to the AU’s Solemn Declaration, its leaders adopted the “Silencing the Guns” initiative, one of the flagship projects of the wider developmental blueprint “Agenda 2063.” The campaign seeks to make 2020 a year of action and mobilization, inspiring all stakeholders to prioritize efforts on peace and effective socio-economic development.

By fitting coincidence, this book is published in the same year—a year which will be forever linked not (seemingly) with peace and security but, instead, the COVID-19 pandemic, which has disrupted life in previously unthinkable ways across all continents. The quest to silence the guns, and build peace and security in Africa, will continue long after this pandemic passes. We hope that this book’s insights and lessons will make a small contribution to that goal.

## The State of Peacebuilding in Africa

*The State of Peacebuilding in Africa* looks back on over 30 years of key experiences across numerous aspects of peacebuilding and highlights key lessons learned that could be used to entrench sustainable peace on the continent.

Building on the research and activities of the Southern Voices Network for Peacebuilding (SVNP)^3^—a continent-wide network of 22 African civil society, policy, research, and academic organizations that works with the Wilson Center’s Africa Program to bring African knowledge, analyses, and perspectives to U.S., African, and international policy on peacebuilding in Africa—this volume brings together the work of distinguished African and international practitioners, scholars, and decision makers.

 Peacebuilding is a complex and multifaceted endeavor, consisting of many different elements. While some elements are not covered as fully as others, most of the core themes are included in this book: conflict prevention and early warning systems; mediation and conflict management; post-conflict reconstruction; disarmament, demobilization, and reintegration (DDR); human rights and justice; and the role of women, religion, humanitarianism, grassroots organizations, and regional and continental bodies. The thematic chapters are complemented by six country and region case studies on the Democratic Republic of the Congo, Rwanda, Sierra Leone, Sudan/ South Sudan, Mozambique, and the Sahel/ Mali.

The chapter authors were given a common template to work from and asked to address three main questions in the peacebuilding realm in Africa since the end of the Cold War: What changes have occurred in thinking and practice at the thematic or country level? What have been the key lessons learned (good and bad) and best practices to emerge from them? And, what are the top policy options or recommendations you would put forward to policymakers and practitioners working on this aspect of peacebuilding?

In some respects, of course, this is well-trodden territory. The fall of the Berlin Wall in 1989 coincided with changing dynamics of conflict across the world, not least in Africa. A rich literature on peacebuilding arose in this new era—as much out of hope that historic fault-lines might be bridged as fear that states were not equipped to deal with the new threats to peace and stability. The colossal challenges and failings of United Nations (UN) peacekeeping in the 1990s, especially in Rwanda and the former Yugoslavia (Srebrenica), led to the landmark “Report of the Panel on United Nations Peace Operations” (informally known as the Brahimi Report), published in 2000, which outlined the need for enhancing the UN’s capacity to undertake a wide variety of missions. It was followed five years later by the UN Secretary-General’s Report “In Larger Freedom,” which emphasized the synergistic relationship of development, security, and human rights in building peace. More recently, the Independent High-level Panel on Peace Operations (HIPPO) was convened in 2014 by the then-Secretary-General Ban Ki-moon. His thorough review of UN peace operations and the emerging needs of the future, published the following year, popularized the idea of “the primacy of politics.” By which the report meant not that UN missions could end conflict and build peace alone, but that their success rested on being part of a “viable process.” Many of the themes addressed in this volume can be understood as key parts of such a process.

With ever-deeper attention given to conflict resolution by the UN, governments, and the academic community, robust debates have arisen over what terms and definitions fit realities on the ground best. This is not merely an academic concern: the lack of a common vocabulary can fatally undermine peace missions. Where there is vagueness and confusion over terms, one of the book’s contributors writes, contrasting interpretations of mandates by different national contingents in peace operations can always arise.

The concept of “ peacebuilding” has been, to say the least, variously defined. For some, it is one of several distinct activities including: conflict prevention and mediation (e.g., early warning and urgent diplomatic measures); peacemaking (e.g., high-level envoys and summits); peace enforcement (e.g., violent and nonviolent coercive measures), and post-conflict reconstruction (including justice, institution-building, and economic development). All of which, in their own way, contribute to international peace and security. And then there are peacekeepers, who are increasingly mandated—or at least find themselves working—across different realms: protecting civilians, delivering humanitarian assistance, helping to restore the rule of law, even engaging in de facto reconstruction and state-building. Others use the term “ peacebuilding” in an instrumental sense: the means to institutionalize peace, and remove the root causes of conflict.

For the purposes of this volume, peacebuilding is understood in a broad sense, an umbrella term that encompasses the activities highlighted above and some others. If a more precise definition is required, perhaps it is worth recalling another important UN document, Boutros Boutros-Ghali’s *Agenda for Peace* published in 1992, which describes the concept of peacebuilding as

the construction of a new environment… which seeks to avoid the breakdown of peaceful conditions. [Only] sustained, cooperative work to deal with underlying economic, social, cultural and humanitarian problems can place an achieved peace on a durable foundation. Preventive diplomacy is to avoid a crisis; post-conflict peacebuilding is to prevent a recurrence.^4^

With a lens on Africa, this definition provides a reasonably accurate frame for the book.

In a growing and increasingly globalized literature on peacebuilding, what sets this volume apart from most others is the amalgam: of contributors from the grassroots and academia, from the practitioner and policymaking worlds; of African and non-African voices. There is richness to this mix. While this book, and the Southern Voices Network for Peacebuilding, the initiative that gave rise to it, privileges African voices, it also includes leading thinkers from outside the continent who have studied and interrogated peacebuilding, and helped shape peacebuilding policies and concepts in Africa.

## Key Issues and Themes in Peacebuilding in Africa

One of the contributors, *Ibrahim Gambari*, a Nigerian former military leader and scholar-diplomat, brings a wealth of high-level experience outside Africa—as a UN Special Envoy to conflict-scarred states like Cyprus and Myanmar—to his reflection on how to build sustainable peace in his home continent. Others, like former government minister *Betty Bigombe*, draw on years as a mediator in her native Uganda—experience that included face-to-face negotiations with Joseph Kony and his Lord’s Resistance Army (LRA)—together with time spent at think tanks and the World Bank to explain why policies on the reintegration of ex-combatants into communities need a rethink. *Vera Songwe*, current Executive Secretary of the United Nations Economic Commission for Africa (UNECA) and a leading African economist and banking executive, draws on the lessons learned from World War One and Two to argue for earlier sequencing of economic development within peacebuilding frameworks, and for revisiting the role of multilateral institutions.

Readers of this volume will invariably pick up on certain issues that percolate across the thematic chapters and case studies. As you progress through *The State of Peacebuilding in Africa*, these are some of the recurrent themes that we think merit closer scrutiny.

1. ***Frameworks.*** Frequently peacebuilding frameworks are out of sync with realities and needs on the ground. Too often this is due to too numerous, various, and uncoordinated—however well-meaning—“partners.” Nor do “one-size-fits-all” approaches work; context matters. Post-conflict reconstruction approaches tend to focus on rebuilding the state while neglecting the reconstruction and healing of the people traumatized by conflict. Both are necessary.
2. ***Mandates and Missions.*** Overloaded mandates—often a laundry list of tasks without commensurate resources in terms of personnel, finances, or logistics—compromise peacebuilding outcomes. Additionally, the growing number of blurred missions—e.g., between humanitarian missions and military peace support operations—is problematic.
3. ***Funding.*** Being overly reliant on external/non-African donors to fund peacebuilding renders key programs vulnerable and unsustainable, as resources are often short-term while peacebuilding needs are long term in nature.
4. ***Civil Society, Grassroots, and Elites.*** Wars are ended by elites; peace is built and sustained at the grassroots. Peace Agreements and peacebuilding efforts need to better reflect that reality, including on matters of transitional justice and on the role played by religion and local infrastructures for peace in African societies. Simply put, local ownership matters and is key to building peace.
5. ***Women’s Voices.*** Peace cannot be realized or sustained if women’s voices are not included in peacebuilding processes or if issues of sexual and gender-based violence are not addressed within peacebuilding efforts.
6. ***Youth.*** Despite recurrent claims to the contrary by leaders and mediators, young Africans are regularly excluded from peace processes. It is still common for youth to be perceived as potentially dangerous “factors” in peacebuilding, easily manipulated to further one or other side’s interests. In reality, young people have agency and are not monolithic actors. They have tremendous potential for driving positive change in Africa where, not infrequently, stale gerontocracies dominate. That soon a quarter of the young people in the world will be African amplifies the need to reimagine the role of youth in peacebuilding.
7. ***Institutions matter: International, African Union, and Regional Economic Communities.*** Peacebuilding achievements should be recognized and built upon. In some cases, core institutions need to revisit their dogmatic approach to peacebuilding. In other cases, the right institutions and processes are in, or being put in place; they just need to be more effective and realized.
8. ***Expectations.*** Peacebuilding is a long-term process, subject to reversals. Potential points of failure are numerous. Too often populations are promised miracles and panaceas. When these do not materialize, resentment can fuel a return to war. Similarly, the international community often expects a sustainable peace but is usually unable or unwilling to make the long-term investment necessary to transform conflict-prone societies.

## Organization of the Book

This book is divided into four parts: (i) peacebuilding in transition in Africa; (ii) strategies and tools; (iii) regional and international dimensions; and (iv) country/region case studies.

Part I focuses on the evolution of peacebuilding and begins with a reflection by *Paul Williams* on more than fifty peace operations deployed to nearly twenty African countries during the twenty-first century. He outlines why peace operations need to be part of a viable strategy of conflict resolution and explains what happens when means and ends are not aligned. Of particular note is his warning that a numbers-centric approach to force generation in peace operations is far less effective than a capabilities and effects-based approach. *Vera Songwe* looks at the economic dimension of peacebuilding reflecting on the failures and successes of international organizations during the first half of the 20th century. She questions the established approach of engaging in economic development only after peace has been restored. This approach, she argues, condemns peacebuilding to failure even before it has started. *Ludovic Lado* examines a vital but under-appreciated factor in the success or failure of peacebuilding: religion. Against a complex and evolving religious landscape, where Christianity and Islam coexist alongside African traditional religions, *Lado* explores the intersection of secular and faith-based processes of peacebuilding, with particular reference to the marginalized role of Muslim-based initiatives. In her chapter on the social imperatives of disarmament, demobilization, and reintegration (DDR), *Betty Bigombe* draws heavily on her leadership experience of DDR initiatives in Uganda and Burundi, highlighting the myriad ways in which greater attention to war’s forgotten noncombatants is essential to heal societies, foster reconstruction and development, and prevent a recurrence of conflict. Similarly, drawing on his own vast experience of UN-led peacekeeping operations in Africa, *Ibrahim Wani* discusses missions’ engagement on issues related to human rights and the protection of civilians. He argues that insufficient political support and overloaded peacekeeping mandates have led to a situation in which human rights and the protection of civilians are not prioritized as highly as they should be. It is thus essential, *Wani* observes, for the UN and its member states to move beyond rhetoric to genuine implementation of the HIPPO framework.

The chapters in Part II explore some of the main tools and strategies used in African peacebuilding. *Lisa Sharland* provides a seemingly obvious but necessary reminder: peacebuilding is less likely to succeed without the participation and consideration of women. In a detailed review of two contrasting cases, Liberia and South Sudan, she reveals some of the challenges and opportunities that UN engagement has offered in terms of advancing equality and women’s security in each country. As overlooked as women, historically, local peace committees have made enormous contributions to peacebuilding in Africa, as *Fritz Nganje* explains. His chapter charts the recent “local turn” which has given rise to diverse forms of grassroots peacebuilding initiatives. Returning to DDR, *Anatole Ayissi* finds that in the vast majority of Africa’s conflict-affected societies, reintegration remains the Achilles Heel of DDR programs; only a minority of ex-combatants are sustainably reintegrated into their communities. He calls for a strengthening of Africa’s ownership of DDR programs, endowing regional institutions with more capacity, expertise, and resources. The thorny subject of African elections is the focus of *Franklin Oduro*’s chapter. He explains why elections, particularly ones that transition societies from autocracy to democracy, are one of the central pillars of peacebuilding in Africa. His chapter concludes with some provisional ideas for mitigating the “winner-takes-all” ethos and other potential triggers for election-related violence in Africa. The last chapter of Part II assesses the contribution of early warning systems to the African Peace and Security Architecture (APSA), with reference to the experience of the West Africa Network for Peacebuilding (WANEP). The authors *Chukwuemeka Eze* and *Osei Baffour Frimpong* argue that WANEP’s work on early warning—and conflict-related early warning systems in general—will not be able to fulfill its potential without reforms in the areas of funding, partnerships with civil society organizations, and closing the chasm between early warning and early response.

Part III highlights the varied regional and international dimensions to African peacebuilding. *Gilbert Khadiagala* commends the African Union for the significant strides the organization has made in building norms around peace, security, stability, and governance, but warns of tremendous obstacles to realizing the vision and objectives articulated in its Post-Conflict Reconstruction and Development (PCRD) policy. He urges national ownership of peacebuilding as well as a deepening and advancing of normative frameworks among various stakeholders. At the Regional Economic Community (REC)-level, the role of the Southern African Development Community (SADC) in attempting to lay the groundwork for peaceful transformation in its region is the subject of *Dimpho Deleglise*’s chapter. In reviewing the cases of SADC mediation and involvement in Lesotho, Madagascar, and Zimbabwe, she explains why the organization has been singularly unable to fulfill its long-term agenda for sustainable peace. *Phil Clark* examines the International Criminal Court (ICC) and its intersections with two widespread domestic conflict resolution processes in Africa: national amnesties and peace negotiations. In doing so, he connects to two overarching scholarly and policy debates, namely the appropriateness and legality of amnesties as opposed to prosecutions for suspected perpetrators of international crimes, and the “peace versus justice” debate over whether the threat of prosecution imperils peace negotiations that involve high-level atrocity suspects. A practitioner’s perspective on the changing role of humanitarian organizations in Africa’s conflict zones is provided by *Jens Pedersen.* He traces how humanitarianism has become a highly contested space on the battlefield, where principles of humanitarian relief have been undermined by the major powers and the UN in their pursuit of ostensibly noble objectives. A different kind of firsthand perspective is offered by *Ibrahim Gambari*. His focus is the prevention and mediation of conflicts, drawing on his experience as a senior UN envoy to several conflict zones around the world. His is a global view on lessons learned for peacebuilding in Africa against the backdrop of fundamental shifts in the nature of conflict since the end of the Cold War.

Part IV of the book captures some of the above peacebuilding themes in several country and region studies. *Rachel Sweet* tackles one of the continent’s most complex and conflict-prone states, the Democratic Republic of the Congo, in a comparative look at two major intervention attempts of the UN Peacekeeping mission in the Democratic Reublic of the Congo (MONUSCO) in different theaters of conflict in North Kivu: one that was seen as a success (against the M23 rebellion, 2012–2013), and the other a failure (against the Allied Democratic Forces [ADF] rebellion, 2014–present). In his detailed account of Mozambique’s decades-long, often fraught peacebuilding journey, *Alex Vines* examines the diverse initiatives—from financial and diplomatic to the contribution of church-based mediation and grassroots initiatives for justice and reconciliation—that brought the ruling FRELIMO party and RENAMO to a negotiated settlement, against a backdrop of changing regional and international dynamics. *Adekeye Adebajo* sets out in his chapter to solve a mystery: why has Sierra Leone remained relatively stable 14 years after peacekeepers left the country in 2006, and 18 years after the end of a devastating 11-year civil war in which an estimated 70,000 people died? In doing so, he explains, Sierra Leone has defied the fate of so many fragile and conflict-prone states. The main departure point of *Jok Maduk Jok’*s chapter on Sudan/ South Sudan is that, common to most protracted conflicts that relapse into war, there was a profound disconnect between elites and local communities in the once-unified Sudan and subsequently in the two separate countries. Drawing heavily on the apparent failures of the Comprehensive Peace Agreement (CPA), he asserts that African conflict resolution and peacebuilding relies too heavily on political agreements between politico-military elites. *Terence McNamee* outlines why Rwanda, a relatively unknown country until 1994, divides opinion among scholars and commentators as perhaps no other state in Africa, if not the world, does. Is it a development success, rising from the ashes of mass ethnic slaughter? Or a case of autocratic recidivism, masked by a bogus narrative of national unity? This chapter tries to find a balance in Rwanda’s highly contested peacebuilding journey. The last chapter, by *Paul Melly*, tracks the evolution of local and international efforts to contain the multifaceted threats to peace and security in the Sahel—threats that have become more serious over the past 15 years, despite a steady reinforcement of the national, regional, and international campaign to stabilize the region.

It is our hope that in addressing peacebuilding in Africa from such varying angles and perspectives, this book surfaces insights and lessons that are useful for strengthening the understanding and practice of peacebuilding in Africa.

### Notes

1. See https://au.int/en/documents/20130613/50th-anniversary-solemn-declaration-2013.
2. See Paul Melly, Chapter 10.1007/978-3-030-46636-7_22 in this book.
3. The Southern Voices Network for Peacebuilding was established in 2011, and is generously funded by the Carnegie Corporation of New York. More information about the SVNP can be found here: https://www.wilsoncenter.org/the-southern-voices-network-for-peacebuilding.
4. See A/47/277-S/24111 June 17, 1992, ‘An Agenda for Peace: Preventive diplomacy, peacemaking and peace-keeping’, the Report of the Secretary-General pursuant to the statement adopted by the Summit Meeting of the Security Council on January 31, 1992.

